# Metagenomic Insights Into the Microbial Assemblage Capable of Quorum Sensing and Quorum Quenching in Particulate Organic Matter in the Yellow Sea

**DOI:** 10.3389/fmicb.2020.602010

**Published:** 2021-01-15

**Authors:** Ying Su, Yuanzhi Yang, Xiao-Yu Zhu, Xiao-Hua Zhang, Min Yu

**Affiliations:** ^1^College of Marine Life Sciences, Institute of Evolution & Marine Biodiversity, Ocean University of China, Qingdao, China; ^2^Laboratory for Marine Ecology and Environmental Science, Qingdao National Laboratory for Marine Science and Technology, Qingdao, China; ^3^Zhongshan School of Medicine, Sun Yat-sen University, Guangzhou, China; ^4^Department of Horticulture and Landscape Architecture, Purdue University, West Lafayette, IN, United States; ^5^Frontiers Science Center for Deep Ocean Multispheres and Earth System, Ocean University of China, Qingdao, China

**Keywords:** quorum sensing, quorum quenching, particulate organic matter, metagenome, microbiota

## Abstract

Quorum sensing (QS) is a density-dependent communicating mechanism that allows bacteria to regulate a wide range of biogeochemical important processes and could be inhibited by quorum quenching (QQ). Increasing researches have demonstrated that QS can affect the degradation of particulate organic matter (POM) in the photic zone. However, knowledge of the diversity and variation of microbial QS and QQ systems in sinking POM is scarce. Here, POM samples were collected from surface seawater (SW), bottom seawater (BW), and surficial sediment (SS) in the Yellow Sea of China. 16S rRNA gene amplicon and metagenome sequencing were performed to analyze the community structure of particle-associated microorganisms and distribution of QS genes [acylated homoserine lactone (AHL) synthesizing gene *luxI* and AHL sensing gene *luxR*] and QQ genes (genes encoding for AHL lactonase and acylase) in POM. Shifting community structures were observed at different sampling depths, with an increase of microbial abundance and diversity from SW to BW. Along with the variation of microbial communities, the abundances of *luxI* and *luxR* decreased slightly but were restored or even exceeded when POM arrived at SS. Comparatively, abundances of AHL lactonase and acylase remained constant during the transportation process from SW to BW but increased dramatically in SS. Correlation tests indicated that abundances of *luxI* and *luxR* were positively correlated with temperature, while those of AHL acylase were positively correlated with depth, SiO_4_^2–^, PO_4_^3–^, and NO_3_^–^, but negatively correlated with temperature and pH. According to phylogenetic analyses, the retrieved QS and QQ genes are more diverse and distinctive than ever experimentally identified. Besides, the vertical transmission of QS and QQ genes along with POM sinking was observed, which could be one of the key factors leading to the prevalence of QS and QQ genes in marine ecosystems. Overall, our results increase the current knowledge of QS and QQ metabolic pathways in marine environment and shed light on the intertwined interspecies relationships to better investigate their dynamics and ecological roles in POM cycling.

## Introduction

Particulate organic matter (POM) is prevalent in marine environment and believed to be the main vehicle for vertical material transport in the ocean (Fowler and Knauer, 1986). They are the foundation of the marine food web and primary food sources for creatures living in the aphotic zone (Azam and Malfatti, 2007). POM are composed of diverse concentrated organic components including pellets, phytoplankton and zooplankton debris, plant secretions, microorganisms, and part of inorganic elements such as dust and detritus (Alldredge and Silver, 1988) that attract quantities of microorganisms to colonize. In return, hydrolytic enzymes secreted by POM-associated microorganisms accelerate the degradation of POM (Smith et al., 1992; Kiørboe et al., 2002). Previous research has revealed that bacterial abundances, community diversities, and the extracellular enzyme activities (EEAs) were higher in POM compared to those in ambient seawater (Smith et al., 1992). The upregulated EEAs in POM were firstly explained using a bacterial density-dependent regulatory mechanism, named quorum sensing (QS), by Hmelo and his colleagues (Hmelo et al., 2011).

Quorum sensing triggers synchronous expression of multiple genes in a microbial population, initiating a coordinated action when high cell densities are reached, e.g., flagella formation (Zan et al., 2012), biofilm formation (Sun et al., 2015), and extracellular enzyme (EE) production (Jatt et al., 2015; Su et al., 2018). QS-based communications are performed based on the production, release, and population-wide detection of several types of QS signaling molecules, named autoinducers (AIs). To date, more than 200 AIs have been identified from a variety of bacteria (Rajput et al., 2016), most of which were classified as types of *N*-acyl homoserine lactones (AHL) (Williams, 2007; Case et al., 2008). AHL molecules have been detected and extracted from various marine environments, such as POM (Hmelo et al., 2011), the *Trichodesmium* phycosphere (Van Mooy et al., 2012), and microbial mats (Decho et al., 2009), implying a prevalence of AHL-based QS in marine ecosystems.

AHL-mediated QS is the best characterized QS system and is commonly found in Gram-negative bacteria (Case et al., 2008; Papenfort and Bassler, 2016). AHLs are produced by LuxI-type AHL synthases and sensed by LuxR-type receptors. Most AHL-producing bacteria that have been isolated from POM belong to *Rhodobacterales*, *Sphingobacteriales*, and *Vibrionales* (Gram et al., 2002; Doberva et al., 2015; Su et al., 2018). Nonetheless, the abundance and diversity of microbial species possessing QS systems in marine environments are greatly underestimated since most of marine microorganisms have not been isolated and cultured. With the employment of metagenomic techniques, novel AHL synthases have been gradually discovered in other clades of bacteria, including the Gram-positive *Exiguobacterium* spp. (Muras et al., 2018) and nitrite oxidizing bacteria *Nitrospirae* spp. (Nasuno et al., 2012). Metagenome analyses, which avoid cultivation biases, have great probability to find new QS systems in marine environments.

Bacterial QS signaling can be disrupted by another mechanism, which is termed quorum quenching (QQ). QQ-based mechanisms are diverse, for example, inhibition of signal reception by secreting inhibitors or antagonists (Manefield et al., 2002) and enzymatic hydrolysis of AIs by QQ enzymes (Dong and Zhang, 2005). Synthesizing QQ enzymes is a popular method for bacteria to interfere with QS-mediated processes, and AHL degradation enzymes have been extensively studied. Two types of AHL degradation enzymes (AHL lactonase and AHL acylase) have been identified so far. The lactonases hydrolyze the HSL ring of the AHL molecule generating the corresponding acyl homoserines (Dong et al., 2007), while the acylases cleave the AHL amide bond to generate the free fatty acid and HSL ring (Romero et al., 2008). QQ has been recommended as a promising strategy for anti-virulence therapy, since it only inhibits QS-regulated virulence instead of cell growth and division, which results in little selective pressure for the evolution of resistance (Tang and Zhang, 2014; Tang et al., 2015). Until now, the most studied QQ enzymes are originated from genus *Bacillus* (Dong et al., 2002; Lee et al., 2002), *Agrobacterium* (Zhang et al., 2002; Uroz et al., 2009), and *Pseudomonas* (Huang et al., 2003; Sio et al., 2006), which are mainly isolated from soil. In contrast, only a few novel QQ enzymes have been identified from marine environments (Tang et al., 2015), which harbor unprecedented diverse microorganisms.

In recent years, the existence and roles of QS and QQ in marine environments have been gradually verified. Diverse QS and QQ bacteria have been isolated from POM (Jatt et al., 2015; Su et al., 2018), corals (Gram et al., 2002; Golberg et al., 2011), dinoflagellates (Wagner-Dobler et al., 2005), and the *Trichodesmium* phycosphere (Van Mooy et al., 2012), while novel QS and QQ genes have been found using metagenomic techniques. According to Doberva and colleagues, genes coding for LuxI, AHL lactonase, and AHL acylase homologs were found in all samples obtained from the Global Ocean Sampling (GOS) database, which implied a prevalence of QS and QQ in the global ocean (Romero et al., 2012; Doberva et al., 2015). Nevertheless, due to the limitation of GOS sampling, little is known about QS and QQ metabolic pathways in vertical distributed POM from Chinese marginal seas.

In the present study, the microbial communities associated with POM were surveyed based on 16S rRNA gene and metagenome sequencing. Furthermore, metagenomic sequencing and analyses were conducted to analyze the abundances and diversities of QS and QQ genes in vertical distributed POM. Our results here expand the current knowledge of QS and QQ metabolic pathways in sinking POM, and help to understand the intertwined interspecies communications in marine ecosystems.

## Materials and Methods

### POM Sampling and Environmental Characterization

Experimental samples were collected in July 2016 onboard the R/V “*Dong Fang Hong* 2” at sampling sites HS5 (121.67° E, 35.50° N) and H12 (124.00° E, 35.00° N) from the Yellow Sea of China (YS) (Supplementary Figure 1). POM suspended at SW and BW were collected using a CTD Rosette sampler (Conductivity–Temperature–Depth, Sea-Bird SBE911) and then size fractioned using 3-μm and 0.22-μm polycarbonate membranes (GTTP, 142 mm, Millipore). The POM sinking to SS were collected via a grab sampler, and only the top layers (≤0.5 mm) were collected using autoclaved knives. Ten samples [HS5-swp, HS5-swf, HS5-bwp, HS5-bwf, HS5-ss, H12-swp, H12-swf, H12-bwp, H12-bwf, and H12-ss; “swp(f),” “bwp (f),” and “ss” represent POM samples from SW, BW, and SS; letters “p” and “f” represent POM samples collected on 3-μm and 0.22-μm polycarbonate membranes, respectively] were obtained and stored in liquid nitrogen onboard and at −80°C in the laboratory until later DNA extraction. In addition, physicochemical parameters of seawater (temperature, salinity, and depth) were recorded with the CTD system. Other environmental factors, including concentrations of ammonium nitrogen (NH_4_^+^), nitrate nitrogen (NO_3_^–^), nitrite nitrogen (NO_2_^–^), and phosphate phosphorus (PO_4_^3–^), were measured according to the methods described in previous studies (Murphy and Riley, 1962; Greenberg et al., 1992).

### DNA Extraction and Quantification of 16S rRNA Gene in POM Samples

The DNA of each POM sample was extracted and purified following the CTAB methods described by Zhou (Zhou et al., 1996). Extracted DNA was quantified using a Qubit Fluorometer (Life Technologies, CA, United States). A total of 10 DNA samples were sent to Novogene (Novogene, Tianjin, China) for metagenomic sequencing.

Quantitative PCR (qPCR) was performed as previously described (Fierer et al., 2005) to assess the abundance of microorganisms in POM samples. Briefly, the abundances of bacteria and archaea were quantified using primer sets Eub338F/Eub518R (Fierer et al., 2005) and Arch16F/Arch344R (Liu et al., 2015), respectively. qPCR reactions were performed in triplicate in a 20-μl system including 10 μl of 2 × SYBR Premix Ex Taq II (Takara Bio Inc.), 0.4 μl of 50 × ROX reference dye, 0.2–0.4 μM of each primer, and 2 μl of 1/10 diluted template DNA. The conditions of PCR were set according to Liu et al. (2018). The amplification efficiency for 16S rRNA gene was 0.93, with an *R*^2^ value of 0.99.

### Sequencing and Analysis of 16S rRNA Gene Amplicons

Microbial communities of the 10 POM samples were analyzed by sequencing 16S rRNA gene amplicons. The variable region four (V4) of bacterial and archaeal 16S rRNA genes was amplified using the golay barcoded primer set 515F/806R (Caporaso et al., 2011) and sequenced on a Illumina Hiseq2500 PE250 platform. All the obtained paired-end reads of 16S rRNA gene amplicons were filtered and clustered into operational taxonomic units (OTUs) with a similarity threshold of 97% using UPARSE (Edgar, 2013). Taxonomic assignment of each OTU’s representative sequences was performed using Mothur (Schloss et al., 2009) against the SILVA databases (Quast et al., 2012). The abundance of each OTU was calculated by summing the abundances of genes annotated to it. The 10 libraries were rarefied to an even depth based on the smallest sample. After the rarefaction, alpha diversities such as Chao 1’ (Chao, 1984), Shannon indexes (Shannon and Wiener, 1963), Simpson indexes (Simpson, 1949), and Good’s coverage were calculated (Supplementary Table 1).

### Metagenomic Sequencing, Assembly, and Annotation

Illumina paired-end libraries were constructed using NEBNext Ultra DNA Library Prep Kit following the manufacturer’s recommendations. Resulting libraries were sequenced on one 2 × 150 bp lane of the Illumina Hiseq X-Ten platform by Novogene (Novogene, Tianjin, China).

For each metagenomic dataset, clean reads with high quality were obtained and assembled into contigs using MEGAHIT (version 0.3.3) (Li et al., 2015) with default parameters but a minimum length of 500 bp. The unemployed paired-end reads from the first fun of MEGAHIT were recycled and used for assembling into contigs again (Qin et al., 2010; Karlsson et al., 2012). Open reading frames (ORFs) were predicted based on assembled contigs using MetaGeneMark (Zhu et al., 2010) with a minimum length of 100 nt. A non-redundant gene catalog was constructed using CD-HIT (Li and Godzik, 2006) with thresholds of 95% identity and 90% coverage. General information for metagenomic data is provided in Supplementary Table 2.

Gene annotation was performed using the BLASTP command implemented in DIAMOND (Buchfink et al., 2015) with a maximum *e*-value of 10^–5^ against the NCBI non-redundant protein database. The taxonomic assignment of each unigene was performed based on the result of gene annotation using MEGAN4 with LCA algorithm (Huson et al., 2011). To calculate the relative abundance of each gene, the number of reads assigned to a specific gene was divided by the length of the gene and subsequently compared to the sum of divided reads number of all genes using the SOAPaligner program (Karlsson et al., 2012; Cotillard et al., 2013).

### Retrieval of Genes Involved in QS and QQ From the POM Metagenomes

Sequences of ratified RecA, LuxI, LuxR, AHL lactonase, and AHL acylase sequences from Swiss-Prot and UniProtKB were used as training protein sequences to create reference profiles. Each profile was aligned using Clustal W (Larkin et al., 2007) in MEGA v.5.1 (Tamura et al., 2011) to discard redundant and partial sequences (Supplementary Table 3). Putative proteins related to QS and QQ in the POM metagenomes were retrieved using a BLASTP search against each reference profile with a cutoff of *e*-value ≤ 1e^–5^. All retrieved homolog sequences were then analyzed with Conserved Domain Database (CDD) at NCBI (Marchler-Bauer et al., 2011)^1^. Candidate sequences were removed if no conserved domains similar to reference proteins was identified.

Relative abundance of each QS or QQ gene was determined by a sum of the abundances of all sequences affiliated to the specific gene and then normalized by the abundance of *recA*, which is a single copy housekeeping gene in most bacteria. The normalized abundance represented the averaging copies of QS or QQ genes in an individual cell.

### Phylogenetic Analyses

The top 25 AHL synthase, AHL receptor, AHL lactonase, and AHL acylase from SW, BW, and SS were selected for subsequent phylogenetic analyses. Both retrieved sequences from POM metagenomes and referring sequences from Swiss-Prot were aligned together using MAFFT v.7.0 (Katoh and Standley, 2013) and adjusted manually. Phylogenetic trees were constructed using IQ-TREE based on maximum likelihood (ML) algorithm with 1000 bootstrap replicates (Nguyen et al., 2014).

### Statistical Analyses

PERMANOVA was used to analyze the correlation between abundances of QS and QQ genes with environmental parameters. Analyses were performed in R using the *adonis* function in the vegan package (Mackelprang et al., 2017). A distance matrix was built based on the weighted UniFrac method (Lozupone et al., 2006) and hierarchical cluster tree was built using UPGMA (unweighted pair group method with arithmetic mean).

### Data Availability

The sequencing data of 16S rRNA amplicons and metagenome used in this study have been deposited in the GenBank Short Read Archive with accession numbers PRJNA632649^2^ and PRJNA428417^3^, respectively.

## Results

### Physicochemical Characteristics of POM Sampling Sites

Physical and chemical properties of each POM sampling site were assessed (Supplementary Table 4). The temperature (ranged from 9.20 to 23.58°C) and pH (ranged from 7.81 to 8.16) decreased, while the salinity increased from 31.48 to 32.85 psu with the sampling depth. The concentrations of PO_4_^3–^ and NO_3_^–^ were much higher in deeper water than that in SW, especially in bottom water at H12. In contrast, the concentration of NH_4_^+^ decreased with depth slightly from 0.021 to 0.012 μmol/L at H12. A remarkably higher concentration of SiO_4_^2–^ (up to 17.97 μmol/L) was also recorded in bottom water at H12.

### Microbial Abundance and Diversity in POM

**FIGURE 1:**
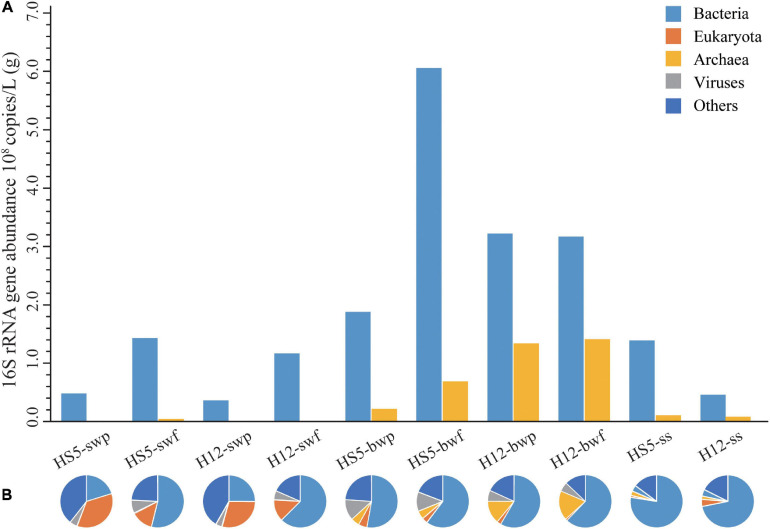
Abundances of bacteria and archaea **(A)** and microbial community structure at phylum level **(B)** in POM.

Great differences of bacterial and archaeal abundances were observed in POM samples at different sampling depth (Figure 1A). Bacterial abundances in SW were about one order of magnitude lower than that in BW samples (Supplementary Table 4, *p* = 0.025, *F* = 9, *N* = 10), while archaeal abundances increased about two to three orders of magnitude from SW to BW (Supplementary Table 4, *p* = 0.019, *F* = 9, *N* = 10). The bacterial and archaeal abundances were higher in smaller size particles (0.22–3 μm) than those in larger particles (>3 μm) from the same sampling layer.

Microbial diversity of the 10 POM samples also exerted differences at different depth. As is shown by alpha indexes, microbial diversities were highest in SS, followed by BW and SW (Supplementary Table 1 and Supplementary Figure 2), which demonstrated an increasing trend with depth.

### Microbial Community Structure in POM

Based on the composition and abundance of OTUs, all the samples were clustered into three groups, SW, BW, and SS, according to UPGMA analysis (Supplementary Figure 3), and the community structures were similar in POM of different size from the same sampling depth. According to the metagenomic analyses, eukaryotes were more representative in SW (13–35%) than in BW (1–5%) and SS (ca. 2%). The relative abundance of bacteria increased from an average of 40% in SW and 58% in BW to 75% in SS. Archaea had the highest relative abundance in BW (11%) compared to that in SW (0.2%) and SS (2%). Viruses accounted for a small proportion in POM collected from SW (3–8%) and SS (ca. 4%) but were abundant in POM collected from BW at HS5 (ca. 12%). Except for that, large proportions of microorganisms were unclassified in SW (31%), BW (19%), and SS (16%) (Figure 1B).

**FIGURE 2:**
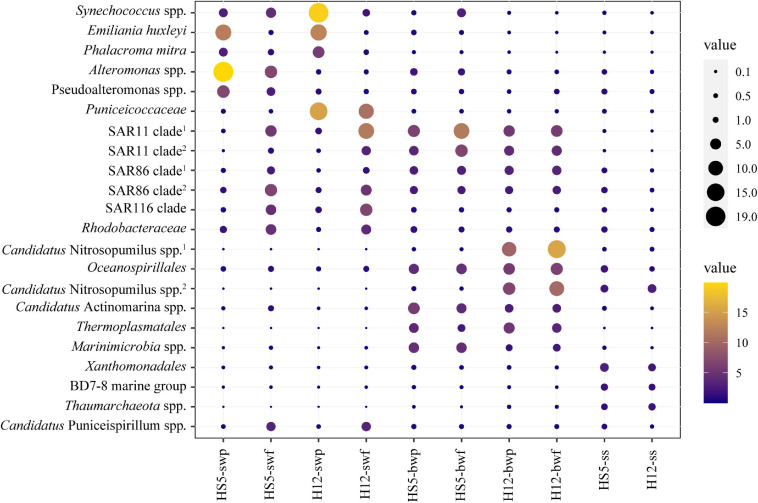
Relative abundances of the top 10 bacteria/archaea within POM communities from surface water, bottom water, and surficial sediment.

Different microorganisms were enriched in POM samples obtained from different depths. In surface water, some eukaryotic phytoplankton (e.g., *Emiliania huxleyi* and *Phalacroma mitra*) and cyanobacteria (e.g., *Synechococcus* spp.) were predominant in POM (especially in particles larger than 3 μm) (Figure 2). Microbial species that belonged to SAR11 clade, SAR86 clade, SAR116 clade, and *Rhodobacteraceae* were also enriched in SW, but more enriched in smaller size particles, i.e., particle size of 0.22–3 μm (Figure 2). Except for that, certain specific species arose at different sampling sites. *Alteromonas* spp. and *Pseudoalteromonas* spp. were typically enriched in SW at HS5, while bacteria that belonged to *Puniceicoccaceae* were predominant in SW at H12 (Figure 2).

**FIGURE 3:**
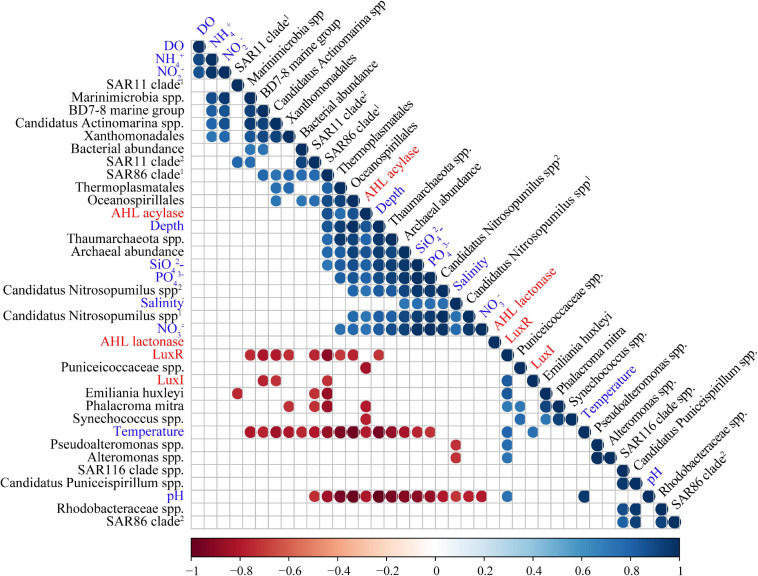
Correlation tests among abundances of QS/QQ genes, environmental variables, and predominant microbial orders. Only those with a *p* value less than 0.05 are indicated by colored circles in the grid. Among them, circles in red represent negative correlations, and circles in blue represent positive correlations. The correlation coefficient is indicated by the color of the color strip below.

Microbial species assigned to SAR11 clade and SAR86 clade remained with high abundance in BW. Moreover, species affiliated with *Oceanospirillales*, *Candidatus* Actinomarina, and *Marinimicrobia* were also distinctive and predominant in BW. Except for bacteria, more abundant archaeal species were identified in BW. *Candidatus* Nitrosopumilus spp. and *Thermoplasmatales* spp. showed higher abundance in BW, accounting for more than 20% in the microbiome at H12 (Figure 2), and were positively correlated with concentrations of SiO_4_^2–^, PO_4_^3–^, and NO_3_^–^ (Figure 3 and Supplementary Table 5).

Species belonging to *Xanthomonadales*, BD7-8 marine group, and *Thaumarchaeota* were distinguished in SS with higher abundance than those in SW and BW (Figure 2). However, several species predominant in SS were also abundant in other groups. For example, species that belonged to *Candidatus* Nitrosopumilus and *Oceanospirillales* were also enriched in BW, while species that belonged to *Rhodobacteraceae*, *Pseudoalteromonas*, and *Candidatus* Puniceispirillum were also enriched in SW. Given that POM is widely accepted as the vehicle transporting materials from surface to seafloor, we have enough reason to suspect that several species colonizing on POM from SS, i.e., *Rhodobacteraceae* and *Oceanospirillales*, were inherited from SW or BW with the transportation of POM.

### Normalized Abundance of QS and QQ Genes in POM

In the POM metagenomes, the normalized abundances of QQ genes encoding for AHL lactonase (2.17) and AHL acylase (0.53) were significantly higher than that of QS genes encoding for AHL synthase (0.02) and AHL receptor (0.53) (Supplementary Table 3). It seems that there existed tremendous species possessing the ability of AHL eavesdropping or AHL degrading ability, rather than synthesizing QS signals.

**FIGURE 4:**
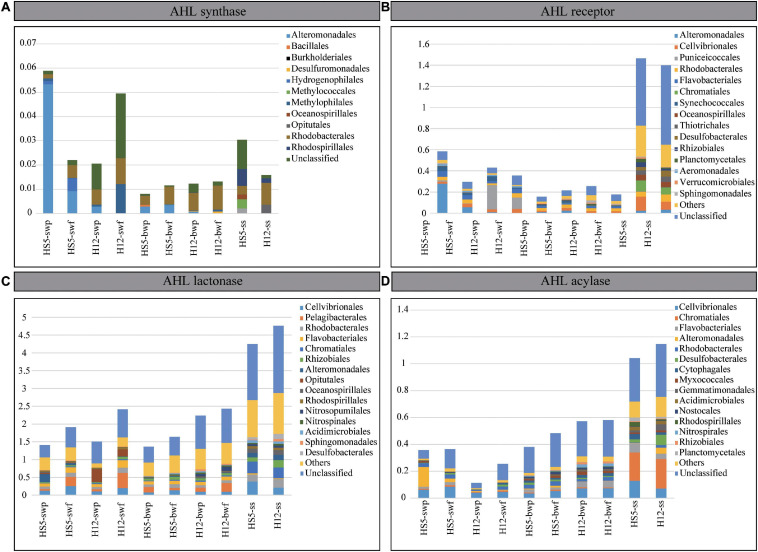
Normalized gene abundances of AHL synthase **(A)**, AHL receptor **(B)**, AHL lactonase **(C)**, and AHL acylase **(D)**. The top 15 microbial orders possessing QS and QQ genes are demonstrated in different colors.

Except for *luxI*, the relative abundances of genes encoding for LuxR, AHL lactonase, and AHL acylase were much higher in POM samples from SS than those from SW and BW (Figure 4 and Table 1). The normalized abundances of *luxR* and two QQ genes in SS were greater than 1.0, and the abundance of AHL lactonase was even higher than 4.0, indicating that every microbial genome in SS contained at least one copy of genes encoding for LuxR, AHL lactonase, and acylase on average. We supposed that the ability perceiving or quenching AHL was more extensive in POM from SS than that from seawater.

**TABLE 1:** Normalized abundance of QS and QQ genes in POM samples.

Sample	LuxI	LuxR	AHL lactonase	AHL acylase
HS5-swp	0.059	0.584	1.298	0.357
HS5-swf	0.022	0.296	1.690	0.365
H12-swp	0.021	0.430	1.528	0.114
H12-swf	0.050	0.355	2.345	0.256
HS5-bwp	0.008	0.156	1.126	0.381
HS5-bwf	0.012	0.216	1.343	0.484
H12-bwp	0.012	0.255	1.895	0.572
H12-bwf	0.013	0.176	2.048	0.581
HS5-ss	0.030	1.464	3.970	1.042
H12-ss	0.016	1.399	4.419	1.148

Compared with the apparent differences in abundances of QS and QQ genes from SS and seawater, the differences of that from SW and BW were less noticeable (Figure 4). In POM from SW, the abundances of encoding genes for LuxI and AHL acylase were higher than that from BW. However, there was no significant difference in the abundance of *luxR* and AHL lactonase encoding genes from SW and BW, which implied that the potential of AHL synthesis decreased along with POM sinking, but the capability of AHL perception or quench remained in POM during the sinking. Indeed, the abundances of the four QS and QQ genes differed between the larger and the smaller size particles from SW. Taking the gene *luxR* as an example, it was more abundant in the larger particles at both sampling sites (Figure 4). Correspondingly, we recognized that the *luxR* from SW were mainly affiliated with *Alteromonadales* and *Puniceicoccales* at HS5 and H12, respectively, which preferred colonizing in larger particles (Figure 2). Therefore, the abundances of QS and QQ genes in different size particles largely depend on the abundances of microorganisms possessing the functional genes. In correspondence with the little discrepancy of microbial abundances in POM with different particle sizes from BW, the differences of QS or QQ genes from different size particles were negligible.

### The Diversity of Microorganisms Possessing QS Genes in POM

A total of 45 LuxI homologs covering 11 bacterial orders were detected from POM samples, most of which belonged to *Proteobacteria* (Figure 4A and Supplementary Figures 4, 5A). In SW, LuxI homologs were mainly found in *Gammaproteobacteria* (*Alteromonadales*), *Betaproteobacteria* (*Hydrogenophilales* and *Methylophilales*), *Alphaproteobacteria* (*Rhodobacterales*), and a large portion of unclassified species. When sinking to BW, the proportion of *Gammaproteobacteria* decreased, but that of *Alphaproteobacteria* (*Rhodobacterales*) held steady. When arriving at SS, more diverse microorganisms, i.e., *Methylococcales*, *Oceanospirillales*, *Opitutae*, and *Rhodospirillales*, were found possessing LuxI homologs, which were quite different from those possessing LuxI from SW and BW.

**FIGURE 5:**
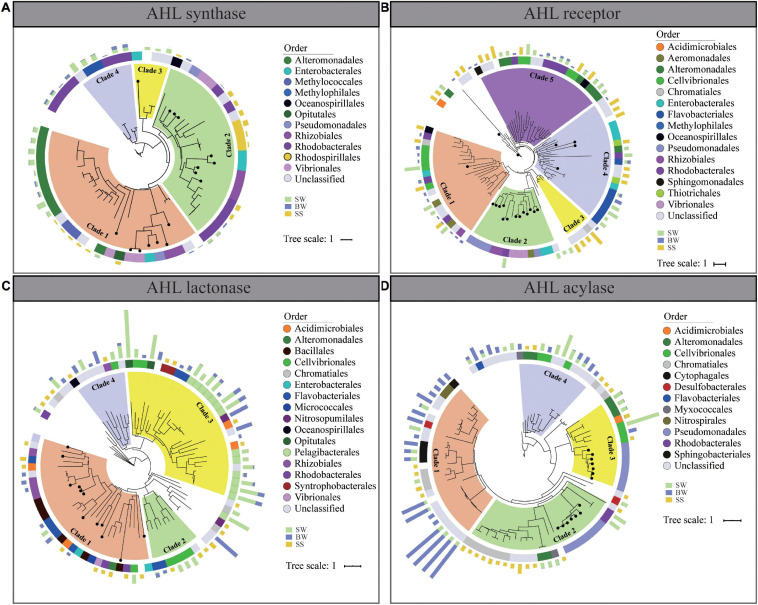
Phylogenetic trees of homologs of AHL synthase **(A)**, AHL receptor **(B)**, AHL lactonase **(C)**, and AHL acylase **(D)** from POM metagenomes. Solid circles on branches mark the identified proteins from Swiss-Prot. The middle color patches represent the taxonomic classification of each sequence on order level. The outer columns with green, blue, and brown colors represent the relative abundances of the corresponding genes in surface water, bottom water, and surficial sediment, respectively.

AHL-perception protein LuxR were more abundant and diverse than AHL-synthesizing protein LuxI in POM. A total of 1070 LuxR homologs affiliating with 70 orders were screened from the metagenomes (Supplementary Table 3 and Supplementary Figure 4). Most of them belonged to bacteria, while a small portion belonged to eukaryotes in SW and archaea in BW (Supplementary Figure 5B). *luxR* genes were prevalent in *Gammaproteobacteria* and *Alphaproteobacteria* in all collected samples. Moreover, the AHL perception was mainly related to *Alteromonadales*, *Puniceicoccales*, *Cellvibrionales*, *Rhodobacteria*, and *Flavobacteriales* in water layers, while microbes possessing LuxR homologs in SS mainly belonged to *Chromatiales*, *Oceanospirillales*, *Thiotrichales*, *Desulfobacteriales*, *Rhizobiales*, and *Planctomycetales* (Figure 4B). Overall, the AHL-based signal perception and transduction existed in more diverse microbial species not merely in *Proteobacteria*.

### The Diversity of Microorganisms Possessing QQ Genes in POM

Quorum quenching genes were more abundant and diverse than QS genes in POM. In the present study, AHL lactonase homologs were found in bacteria, archaea, eukaryotes, and even viruses (Supplementary Figure 5C) covering at least 115 orders (Supplementary Figure 4). Eukaryotes (accounting for 4–16%) with lactonase were mainly found in SW, while archaea (accounting for 8–20%) with lactonase were mainly found in BW, corresponding to the microbial structures in sinking POM (Figure 1). Bacteria with AHL lactonases in SW mainly belonged to *Cellvibrionales, Pelagibacterales*, *Rhodobacterales*, *Flavobacteriales*, and *Opitutales*, and with POM sinking to BW, the proportions of *Rhizobiales* and *Sphingomonadales* increased (Figure 4C). Comparatively, the microbial assemblage possessing AHL lactonase from SS differed from that from water layers, which encompassed more proportions of *Rhodobacterales*, *Chromatiales*, *Oceanospirillales*, *Rhodospirillales*, *Acidimicrobiales*, *Sphingomonadales*, and *Desulfobacterales*.

The abundance and diversity of AHL acylase were much lower than that of AHL lactonase. Around 55 orders were detected possessing AHL acylase (Supplementary Figure 4). Several AHL acylase homologs detected in BW were originated from eukaryotes; however, all of which found in SW were derived from bacteria (Supplementary Figure 5D). Microorganisms possessing AHL acylase in all samples mainly belonged to *Proteobacteria* (e.g., *Cellvibrionales*, *Alteromonadales*, and *Rhodobacterales*), and those enriched in BW also included *Flavobacteriales*, *Myxococcales*, *Nitrospirales*, and *Rhizobiales.* Moreover, microbes possessing AHL acylase from SS also affiliated with other orders, e.g., *Chromatiales*, *Desulfobacterales*, *Gemmatimonadales*, *Acidimicrobiales*, *Rhodospirillales*, and *Planctomycetales* (Figure 4D). Obviously, a wide range of microbial species in POM possessed QQ abilities no matter in water layers or SS.

### Shifts in Phylogenetic Diversity of QS and QQ Genes in POM

Phylogenetic analyses were conducted to assess the diversities of retrieved QS and QQ genes and their homologies to the identified corresponding genes (Figure 5). The protein sequences of AHL synthase could be clustered into four clades. Clade 1 contained sequences mainly affiliated with *Alteromonadales* and *Methylococales*, with the identified AHL synthase from *Vibrionales*, *Rhizobiales*, *Pseudomonadales*, and *Enterobacteriaceae*, while in clade 2, most retrieved AHL synthases affiliated with *Rhodobacterales*, *Rhizobiales*, and *Rhodospirillales* with the identified AHL synthase (Figure 5A).

Protein sequences of AHL receptors and QQ enzymes were clustered into separated clusters, sharing little homologies to their corresponding identified proteins. As for AHL receptor, all sequences could be clustered into five clades, and most of them belonged to *Cellvibrionales* (clade 1 and clade 5), *Flavobacteriales* (clade 4), and *Rhodobacterales* and *Alteromonadales* (clade 5) (Figure 5B). In terms of the two QQ enzymes, protein sequences retrieved from POM metagenomes were evolutionarily distant from the identified QQ enzymes. AHL lactonase was mainly affiliated to *Flavobacteriales* (clade 2 and clade 3), *Cellvibrionales* (clade 2 and clade 3), and *Pelagibacterales* (clade 3) (Figure 5C), while AHL acylase mainly belonged to *Nitrospriales* (clade 1), *Cytophagales* (clade 1), *Chromatiales* (clade 1 and clade 2), *Cellvibrionales* (clade 3 and clade 4), and *Alteromonadales* (clade 3 and clade 4) (Figure 5D). Notably, there was no obvious discrepancy of QS and QQ proteins from different sampling depth in clade clustering. The vertical transmission of QS and QQ genes might occur during the vertical transportation of POM from SW to a deeper area. According to the results of phylogenetic analyses, AHL receptor, AHL lactonase, and AHL acylase from POM were more diverse than previously identified (Figure 5).

### Correlations Between QS/QQ Genes and Environmental Variables

Correlation tests between environmental variables and abundances of QS or QQ genes from different water layers (excluding that of SS for lacking of the environmental parameters) were performed using a Spearman correlation test (discrete quantitative variables, non-normal). LuxI was found to be positively correlated with temperature, while LuxR was found to be positively correlated with temperature, pH, and also the abundance of LuxI accordingly (Figure 3 and Supplementary Table 6). In terms of two QQ genes, AHL lactonase exhibited seldom correlation with environmental variables. However, AHL acylase showed positive correlations with depth, SiO_4_^2–^, PO_4_^3–^, and NO_3_^–^, and negative correlations with temperature and pH (Figure 3 and Supplementary Table 6).

In order to reveal whether the correlations between QS/QQ genes and environmental variables are attributed to certain species with high abundances, the correlations of species enriched in POM microbial communities with both QS/QQ genes and environmental variables were also analyzed. LuxI was negatively correlated with the BD7-8 marine group, *Candidatus* Actinomarina spp., and SAR86 clade (Figure 3 and Supplementary Table 6), but none of them demonstrated correlation with temperature (Figure 3 and Supplementary Table 5). Similarly, *E. huxleyi*, *P. mitra*, *Pseudoalteromonas* spp., and *Alteromonas* spp. that were positively correlated with LuxR did not show positive correlations with temperature or pH (Figure 3 and Supplementary Table 5). Nevertheless, correlations between AHL acylase and environmental variables might be due to several species, i.e., *Thaumarchaeota* spp. and *Candidatus* Nitrosopumilus spp., which also correlated with depth, SiO_4_^2–^, PO_4_^3–^, and NO_3_^–^ (Figure 3 and Supplementary Table 5). We suspect that the environmental condition could affect the distribution and variance of QS genes to a certain extent. However, the results must be carefully interpreted because the correlation might be an artifact due to the abundance of microbes.

## Discussion

In this study, we analyzed the microbial community and the pivotal genes related to QS and QQ mechanisms in the distributed POM collected from the YS. Microbial abundances and diversities increased along with POM sinking from SW to BW, and the diversities reached highest when POM was sinking to SS. However, the abundances of *luxI* and *luxR* even decreased slightly. Except for that, abundances of encoding genes for AHL lactonases and acylases held steadily in water column, but increased dramatically in SS, which indicated higher possibilities of AHL-degradation capabilities in POM from SS. According to the results of phylogenetic analyses, we assumed that the vertical transmission of QS and QQ genes exist in POM, which might be one of the key factors resulting in the high abundance and diversity of QS and QQ genes in POM. Our results add to the current knowledge of QS and QQ metabolic pathways in POM, and shed light on the intertwined interspecies relationships to better investigate their dynamics and ecological roles in POM cycling.

### Different Microbial Assemblage in Vertical Distributed POM

The YS is a typical semi-enclosed marginal sea in China. It is an active area for POM processing and recycling with active land–ocean interaction, which is strongly influenced by rivers, terrestrial input, human activities, and complex current (Lin et al., 2005; Zhu et al., 2018). The sampling sites HS5 and H12 are located at the same latitude, but HS5 is closer to the coast, where it is more affected by terrigenous materials. However, much more discrepancies of microbial communities resulted from variant depth, instead of the horizontal distance (Figure 2 and Supplementary Figure 3). We therefore segregated the 10 samples into three groups (SW, BW, and SS) according to the sampling depth.

Bacterial colonization was more likely impacted by the particle size in SW rather than in other sampling depth. Photoautotrophic Cyanobacteria (*Synechococcus* spp., and *P. mitra*) and *E. huxleyi* were more enriched in larger POM collected from SW (Figure 2) for sufficient light energy and nutrients, which is in coincidence with the previous observations (Cottrell and Kirchman, 2009; Thiele et al., 2015). Conversely, the SAR clusters (including SAR11, SAR86, and SAR116) preferred smaller particles in SW. They were also numerically significant in the Chinese marginal seas, which were of considerable interest to transport and metabolize the POM compounds (Giovannoni and Rappé, 2000). Bacteria belonging to *Rhodobacteraceae* are another predominant member colonizing marine particles (Dang et al., 2008; Geng and Belas, 2010). Many of them possess dual-particle-associated and free-living lifestyles (Geng and Belas, 2010; Zan et al., 2012, 2015), which could be switched by QS-regulated mechanisms, such as flagellar motility and biofilm formation (Zan et al., 2012; Su et al., 2018), antimicrobial indigoidine biosynthesis (Cude et al., 2015), TDA production (Berger et al., 2011), and hydrolytic enzyme production (Su et al., 2018). That might be the reason for the even distribution of *Rhodobacteraceae* on different size particles. Moreover, some specific species, like *Alteromonas* spp. and *Puniceicoccaceae* spp., were predominant at HS5 and H12, respectively. Both of them were pivotal polysaccharide degraders in marine systems with diverse CAZyme repertoires, which play important roles to remineralization of chemically diverse POM (Martinez-Garcia et al., 2012; Koch et al., 2019).

*Oceanospirillales*, *Candidatus* Actinomarina, and *Marinimicrobia* were distinctive and predominant species in BW. Many studies have revealed their roles in carbon utilization (Bull et al., 2005; Cao et al., 2014; De Corte et al., 2018); therefore, they might be the key participants involved in the hydrocarbon utilization in a deeper water layer. Moreover, archaea belonging to *Candidatus* Nitrosopumilus and *Thermoplasmatales* accounted for more than 20% in BW microbiome and positively correlated with concentrations of SiO_4_^2–^, PO_4_^3–^, and NO_3_^–^. Specifically, these archaea might be highly involved in carbon (Poulsen et al., 2013), nitrogen (Lloyd et al., 2013), and sulfur cycling processes (Zhang et al., 2015).

The recalcitrant organic components in POM accumulate with depth during the sinking from SW to seafloor, given that the biodegradable components are continuously consumed by surrounding bacteria. Nonetheless, hydrolysis rates were found highest in surface or near-surface sediments (Meyer-Reil, 1986; Poremba and Hoppe, 1995), which may result from the associated microbial communities with versatile hydrolytic enzymes. *Xanthomonadales*, *Desulfuromonadales*, and *Nitrospirales* were usually observed in areas with adequate organic matter, e.g., coastal seawater (Dang et al., 2011), metal-contaminated soils (Hemmat-Jou et al., 2018), and crude oil field soil (Abbasian et al., 2016), which is in correspondence with results in this study. Moreover, part of dominant microorganisms in SS might be inherited from BW (e.g., *Candidatus* Nitrosopumilus spp. and *Oceanospirillales*), and even from SW (e.g., *Rhodobacteraceae*, SAR86 clade and *Pseudoalteromonas* spp.). The migration of microorganisms along with sinking POM might be a common process in marine ecosystem. Besides, there was no absolutely superior species in SS compared with those in SW and BW, from where the microbial diversities were the highest.

### Distribution and Microbial Composition of QS Genes in POM

Quorum sensing widely existed in marine environments and is of great significance to marine ecosystems by regulating POM degradation (Hmelo et al., 2011; Su et al., 2018), maintaining a healthy and stable state of coral environment (Golberg et al., 2011), and promoting organic phosphorus cycling (Van Mooy et al., 2012). Therefore, revealing the abundance and distribution of QS in POM will facilitate our knowledge of microbial roles in marine environments. In previous studies, the QS systems were mainly characterized from the cultivated bacteria, while lots of information from the uncultivated microorganisms were missed. Here we utilized the metagenomic analyses to reveal the distribution pattern of QS and QQ systems in POM. Genes encoding for LuxI, LuxR, and the two QQ enzymes, AHL lactonase and AHL acylase did not exhibit the highest abundance in the same fraction. LuxI homologs were constrained in bacteria, especially in *Proteobacteria*; thus, the high abundance of LuxI in the surface POM seemed largely dependent on the portion of specific species, such as *Alteromonas* spp. Conversely, LuxR and the two QQ enzymes are more widely distributed in diverse species across bacteria, archaea, viruses, and eukaryotes (Supplementary Table 3); thus, their abundances rely on the structure of microbial community more than a single species. Therefore, the abundance of LuxI is likely to be capricious, but that of LuxR and the two QQ enzymes could resist slight fluctuations caused by succession of microbial community.

To date, genes encoding for LuxI (Doberva et al., 2015), AHL lactonase, and AHL acylase (Romero et al., 2012) have been found ubiquitous in global SW using metagenomic analyses. Nonetheless, the abundance and diversity of QS and QQ genes in marine environment could be more abundant than ever expected, which may result from the vertical transmission of QS and QQ genes along with the sinking of POM.

In this study, we found that the averaging abundance of *luxI* in POM from YS (ca. 0.02) is significantly higher than that in the GOS dataset (0.007). The discrepancy may result from the different sampling sites and screening methods. The GOS metagenomic dataset was mainly collected from the surface water from the Atlantic, Pacific, and Indian Oceans. A wide range of oceans were sampled in GOS project except the marginal seas of China, which possess large input volume of POM and diverse microorganisms. It seems like QS potentials in POM of marginal seas are underestimated. Besides, Doberva and his colleagues used a quite strict selection criteria for sequence screening, and only the *luxI* homologs in *Alphaproteobacteria* were detected (Doberva et al., 2015). Comparatively, we conducted BLASTP with relatively loose parameters, and discarded sequences without functional domains manually to secure the coverage and accuracy of screening. The activities of LuxI homologs have been confirmed in *Actinobacteria*, *Alphaproteobacteria*, and *Gammaproteobacteria* (Jatt et al., 2015; Su et al., 2018), which was in correspondence with the results here. It was verified that the screening method used in this study was reliable for the retrieval of QS genes.

The relative abundance of *luxR* (0.53) was much higher than that of *luxI*, which attributed to quantities of solo *luxR* genes in bacteria (Hudaiberdiev et al., 2015), archaea (Pérez-Rueda et al., 2004), and algae (Figure 3). Furthermore, the abundance of *luxR* in POM from SS was seven times higher than that from seawater, which indicated that microbiota in POM from SS may have greater potential receiving AHL signals to regulate community behaviors. Considering the enormous biological diversity present in an ecological niche, it seems logical that bacteria would produce or receive signals enabling communication with fungi, plants, and animals.

### Distribution and Microbial Composition of QQ Genes in POM

Quorum quenching bacteria have been isolated from both eutrophic marine niches, e.g., POM and *Trichodesmium* phycosphere (Van Mooy et al., 2012) and oligotrophic seas (Romero et al., 2011), which are more universal than QS bacteria in marine environments. It was reported that the abundance of marine cultivable bacteria with QQ activity (Romero et al., 2011) and the frequency of QQ genes in marine metagenomes (Romero et al., 2012) were higher than that of QS bacteria and QS genes. In our previous study, 16 and 51% cultivable species isolated from POM have been experimentally verified possessing AHL synthesizing and degrading activities, respectively (Su et al., 2018). Moreover, in our present study, the abundances and diversities of QQ genes in POM from the YS were also higher than that of QS genes, which was consistent with the previous studies. It was implied that microbial QQ activities were more prevalent in marine environments and might have more important ecological roles than ever expected.

Quorum quenching mechanisms are beneficial to microbial competition by limiting the growth and the coordination of bacteria engaged in QS communication (Defoirdt et al., 2004; Rasmussen and Givskov, 2006), which help keep the homeostasis of microbial communities. Moreover, microorganisms in open sea might use QQ enzymes to degrade AHL signaling molecules for additional energy supply. Therefore, the QQ process could serve as a universal adaptive strategy for microorganisms in marine environment.

The production of virulence factors in most pathogens were regulated by QS systems, such as *Pseudomonas aeruginosa*, *Vibrio harveyi*, and *Legionella pneumophila* (Tiaden et al., 2007; Nackerdien et al., 2008; Jimenez et al., 2012). Therefore, interfering with QS metabolic processes would attenuate their pathogenicity and be developed as new therapies combating pathogens. In recent years, more researches focused on developing novel QQ agents derived from marine environments for combating antibiotic-resistant bacteria in aquaculture, agriculture, and anti-biofouling (Tang et al., 2015; Grandclément et al., 2016). Our results revealed that a large number of QQ strains are unexplored in the POM from the YS, especially in SS, thus provide invaluable information and inspiration for the study of marine-derived QQ agents in the future.

## Conclusion

Microbial abundances and diversities increased along with POM sinking from SW to BW, and the diversities reached the highest when POM was sinking to SS. The abundances and diversities of QS (*luxI* and *luxR*) and QQ (AHL lactonase and AHL acylase) genes varied in seawater and SS. The abundance of *luxI* was the highest in SW, while that of LuxR and two QQ enzymes reached the highest in SS, showing the prevalence of QS signaling and interfering in SS. In addition to bacteria, *luxR* and QQ genes were further detected in archaea and eukaryotes. We assumed that AHL-based QS and QQ might be responsible for cross-kingdom interactions in POM. Our results provide support for future research on microbial cooperation or competition activity, and interaction mechanisms of bacteria in POM. Nonetheless, the presence or absence of QS and QQ genes only suggests the potential of QS and QQ activities in POM, and the expression level of QS or QQ genes and whether the associated reactions are active need further detection. In the future, investigations combining metagenomic, metatranscriptomic, and metabolomic analyses will be performed to restore the expression of QS and QQ genes and their regulatory roles in the biogeochemical cycle.

## Data Availability Statement

The datasets presented in this study can be found in online repositories. The names of the repository/repositories and accession number(s) can be found below: https://www.ncbi.nlm.nih.gov/, PRJNA632649; https://www.ncbi.nlm.nih.gov/, PRJNA428417.

## Author Contributions

YS, MY, and X-HZ designed the experiments. YS and YY analyzed the data, performed the statistical analysis, and wrote the manuscript. MY, X-HZ, and X-YZ revised the manuscript. All authors contributed to manuscript revision and approved the submitted version.

## Conflict of Interest

The authors declare that the research was conducted in the absence of any commercial or financial relationships that could be construed as a potential conflict of interest.

## Footnotes

1 http://www.ncbi.nlm.nih.gov/Structure/cdd/cdd.shtml

2 https://www.ncbi.nlm.nih.gov/bioproject/PRJNA632649

3 https://www.ncbi.nlm.nih.gov/bioproject/PRJNA428417/
