# Supplementary material for: Metagenomic Insights Into the Microbial Assemblage Capable of Quorum Sensing and Quorum Quenching in Particulate Organic Matter in the Yellow Sea
Source: Front Microbiol. 2021 Jan 15;11:602010. doi: 10.3389/fmicb.2020.602010 (PMC7843935; doi:10.3389/fmicb.2020.602010)
Supplement: Supplementary file 1 [file Data_Sheet_1.PDF]

Supplementary Material

Supplementary Tables

Supplementary Table 1. Data of microbial richness and diversity in POM of Chinese marginal seas

Sample name	OTU numbers	Shannon index	Simpson index	Chao 1 index	ACE index	Goods coverage
H12-swp	1351	5.237	0.914	1614.343	1711.013	0.994
H12-swf	1544	6.042	0.958	1945.842	2057.386	0.993
HS5-swp	1532	5.998	0.934	1835.17	1896.947	0.994
HS5-swf	1614	6.735	0.975	1896.62	1978.539	0.994
H12-bwp	2143	6.994	0.971	2676.979	2812.983	0.99
H12-bwf	1649	6.211	0.95	2022.102	2076.639	0.993
HS5-bwp	2339	7.546	0.982	2756.56	2867.537	0.991
HS5-bwf	1957	6.971	0.97	2319.503	2411.64	0.992
H12-ss	5315	10.492	0.997	5615.003	5727.84	0.989
HS5-ss	3673	9.426	0.995	4053.678	4113.687	0.99

Supplementary Table 2. General information of each metagenome

Sample	Raw data	Clean data	ORFs No.[a]	Average length (bp)	GC (%)
HS5-swp	19,150.59	19,029.69	904,676	471.3	55.1
HS5-swf	24,271.98	24,185.83	1,126,140	556.1	50.7
H12-swp	17,275.71	17,146.84	618,594	434.9	53.1
H12-swf	18,151.63	18,102.80	649,512	576.3	45.1
HS5-bwp	20,370.21	20,299.88	862,457	526.4	42.6
HS5-bwf	21,989.78	21,933.21	1,046,683	572.2	45.7
H12-bwp	19,215.45	19,123.78	759,506	525.2	45.6
H12-bwf	17,320.50	17,284.13	1,015,040	544.0	40.9
HS5-ss	27,322.49	27,270.10	1,092,333	511.2	54.9
H12-ss	31,194.02	31,111.29	769,725	481.8	53.4

[a] ORFs No: Number of predicted ORFs (Open Reading Frames).

Supplementary Table 3. Information of reference sequences and retrieval sequences related to QS and QQ

Features \ Protein family	AHL synthase	AHL receptor	AHL lactonase	AHL acylase	RecA
Number of reference sequences	458	972	189	210	577
Average number of amino acids of reference sequences	216	236	292	762	358
Taxonomic affiliation of reference sequences	*Proteobacteria*; *Nitrospirae*	*Proteobacteria*; *Nitrospirae*; FCB group; *Chrysiogenetes*	Archaea; Bacteria; Eukaryota; Metagenomes	Archaea; Bacteria; Eukaryota; Metagenomes	Bacteria; Eukaryota
Conserved domains of reference sequences	pfam00765; COG3916; PRK13834	cl27263; cl21459; cl28474; cl25645; cd06170; pfam00196; pfam03472; COG0745; COG2197; COG2771; smart00421;	cd07729	COG2366	cd00983
Number of retrieved sequences	45	1070	4247	1362	
Normalized abundance of retrieved sequences	0.02	0.53	2.17	0.53	
Taxonomic affiliation of retrieved sequences	*Bacteria*	Archaea; Bacteria; Eukaryota	Archaea; Bacteria; Eukaryota; Viruses	Bacteria; Eukaryota;	

Supplementary Table 4. Physical and chemical properties of POM samples [a, b]

Sampling sites	Depth (m)	Temperature (°C)	pH	DO (mg/L)	Salinity (psu)	PO_4^{3-} (μmol/L)	NO_3^- (μmol/L)	NO_2^- (μmol/L)	NH_4^+ (μmol/L)	SiO_4^{2-} (μmol/L)	Archaea ×10^6 copies/ L(g)	Bacteria ×10^6 copies/ L(g)
HS5-swp	4.0	23.58	8.16	7.42	31.48	0.09	0	0.02	0.18	2.09	0.68	48.07
HS5-swf											5.08	142.68
H12-swp	3.7	23.14	8.12	.7.58	32.38	0.06	0.07	0.02	0.02	1.71	0.39	36.32
H12-swf											0.29	117.44
HS5-bwp	44.0	10.31	7.93	8.09	32.02	0.18	0.19	0.30	1.30	5.05	21.38	187.52
HS5-bwf											68.81	605.91
H12-bwp	74.0	9.20	7.81	7.20	32.85	0.83	12.69	0.05	0.01	17.97	135.01	321.53
H12-bwf											140.48	317.17
HS5-ss	45.0	ND	ND	ND	ND	ND	ND	ND	ND	ND	9.43	139.41
H12-ss	81.0										7.19	46.16

[a] Words "swp(f)", "bwp (f)" and "ss" represent POM from surface, bottom seawater and surficial sediment, respectively; letters "p" and "f" represent POM samples collected on 3 μm and 0.22 μm polycarbonate membranes, respectively.

[b] ND, Not detected.

Supplementary Table 5. Correlation tests between environmental variables and predominant microbial orders [a]

	**Depth**		**Temperature**		**pH**		**DO**		**Salinity**	
**Bacterial order**	*p*-value	ρ	*p*-value	ρ	*p*-value	ρ	*p*-value	ρ	*p*-value	ρ
SAR11 clade[1]	0.642	0.196	0.439	-0.321	0.543	-0.254	0.280	0.436	0.645	0.194
Alteromonas spp.	0.255	-0.457	0.224	0.484	0.173	0.534	0.709	-0.158	0.040	-0.730
Synechococcus spp.	0.188	-0.519	0.194	0.513	0.230	0.479	0.911	0.048	0.899	-0.054
Puniceicoccaceae spp.	0.169	-0.538	0.156	0.552	0.246	0.465	0.959	0.022	0.612	0.213
Emiliania huxleyi	0.149	-0.559	0.127	0.585	0.145	0.565	0.828	-0.092	0.461	-0.306
Candidatus Nitrosopumilus spp[1]	0.014	0.814	0.103	-0.618	**0.024**	**-0.776**	0.102	-0.619	**0.035**	**0.743**
Oceanospirillales spp.	**0.000**	**0.992**	**0.000**	**-0.972**	**0.000**	**-0.989**	0.898	-0.054	0.093	0.632
SAR86 clade[2]	0.516	-0.271	0.473	0.298	0.477	0.296	0.808	-0.103	0.530	-0.263
SAR11 clade[2]	0.057	0.692	0.017	-0.800	**0.037**	**-0.737**	0.356	0.378	0.267	0.447
Candidatus Nitrosopumilus spp[2]	**0.010**	0.836	0.085	-0.644	**0.017**	**-0.799**	0.113	-0.604	**0.032**	**0.751**
SAR86 clade[1]	**0.007**	**0.856**	**0.006**	**-0.864**	**0.009**	**-0.843**	0.964	0.019	0.318	0.406
Candidatus Actinomarina spp.	0.066	0.675	**0.008**	**-0.850**	0.051	-0.705	0.151	0.558	0.711	0.157
Rhodobacteraceae	0.168	-0.539	0.139	0.571	0.140	0.570	0.769	-0.124	0.255	-0.457
SAR116 clade	0.135	-0.576	0.114	0.603	0.147	0.562	0.878	-0.065	0.667	-0.181
Pseudoalteromonas spp.	0.209	-0.498	0.168	0.539	0.136	0.575	0.629	-0.203	0.044	-0.720
Thermoplasmatales spp.	**0.000**	**0.948**	**0.001**	**-0.932**	**0.000**	**-0.948**	0.926	-0.040	0.109	0.610
Marinimicrobia spp.	0.121	0.593	**0.015**	**-0.808**	0.089	-0.638	0.057	0.693	0.806	0.104
Phalacroma mitra	0.094	-0.630	0.078	0.656	0.106	0.614	0.870	-0.070	0.634	-0.200
Candidatus Puniceispirillum spp.	0.209	-0.498	0.179	0.527	0.202	0.505	0.821	-0.096	0.511	-0.274
Xanthomonadales	0.074	0.662	**0.016**	**-0.807**	0.068	-0.672	0.261	0.452	0.822	0.095
BD7-8 marine group	0.127	0.585	**0.026**	**-0.768**	0.114	-0.602	0.142	0.568	0.990	-0.006
Thaumarchaeota spp.	**0.000**	**0.960**	**0.005**	**-0.871**	**0.000**	**-0.945**	0.510	-0.275	0.050	0.706

Bacterial order	PO_4^{3-}		NO_3^-		NO_2^-		NH_4^+		SiO_4^{2-}	
	p-value	ρ	*p*-value	ρ	*p*-value	ρ	*p*-value	ρ	*p*-value	ρ
SAR11 clade[1]	0.976	-0.013	0.893	-0.057	0.259	0.454	0.323	0.402	0.966	0.018
Alteromonas spp.	0.401	-0.346	0.417	-0.335	0.548	-0.251	0.808	-0.103	0.364	-0.372
Synechococcus spp.	0.279	-0.437	0.354	-0.379	0.519	-0.269	0.585	-0.230	0.264	-0.449
Puniceicoccaceae spp.	0.299	-0.421	0.415	-0.336	0.365	-0.372	0.362	-0.373	0.283	-0.434
Emiliania huxleyi	0.291	-0.428	0.362	-0.374	0.385	-0.357	0.515	-0.272	0.263	-0.450
Candidatus Nitrosopumilus spp[1]	**0.000**	**0.957**	**0.000**	**0.964**	0.628	-0.204	0.379	-0.361	**0.000**	**0.950**
Oceanospirillales spp.	**0.007**	**0.853**	**0.022**	**0.782**	0.290	0.428	0.523	0.267	**0.004**	**0.881**
SAR86 clade[2]	0.656	-0.188	0.694	-0.166	0.631	-0.202	0.739	-0.141	0.629	-0.203
SAR11 clade[2]	0.299	0.421	0.412	0.338	0.076	0.658	0.171	0.536	0.246	0.464
Candidatus Nitrosopumilus spp[2]	**0.000**	**0.969**	**0.000**	**0.973**	0.672	-0.179	0.411	-0.339	**0.000**	**0.963**
SAR86 clade[1]	0.054	0.699	0.100	0.621	0.252	0.459	0.408	0.341	0.041	0.727
Candidatus Actinomarina spp.	0.454	0.311	0.650	0.192	**0.004**	**0.877**	**0.017**	**0.800**	0.378	0.362
Rhodobacteraceae	0.320	-0.405	0.377	-0.363	0.404	-0.344	0.575	-0.235	0.288	-0.430
SAR116 clade	0.276	-0.440	0.360	-0.375	0.350	-0.382	0.445	-0.316	0.250	-0.461
Pseudoalteromonas spp.	0.383	-0.359	0.413	-0.338	0.442	-0.319	0.690	-0.169	0.342	-0.389
Thermoplasmatales spp.	**0.015**	**0.810**	**0.035**	**0.742**	0.302	0.419	0.528	0.264	**0.009**	**0.838**
Marinimicrobia spp.	0.662	0.185	0.886	0.061	0.000	0.956	**0.003**	**0.889**	0.565	0.241
Phalacroma mitra	0.222	-0.486	0.305	-0.417	0.317	-0.407	0.417	-0.335	0.198	-0.509
Candidatus Puniceispirillum spp.	0.366	-0.371	0.438	-0.321	0.415	-0.337	0.534	-0.260	0.336	-0.392
Xanthomonadales	0.409	0.341	0.588	0.227	**0.020**	**0.789**	**0.042**	**0.725**	0.345	0.386
BD7-8 marine group	0.594	0.224	0.810	0.102	**0.005**	**0.868**	**0.012**	**0.822**	0.513	0.273
Thaumarchaeota spp.	**0.001**	**0.926**	**0.004**	**0.883**	0.639	0.198	0.942	0.031	**0.000**	**0.942**

[a] The *p*-values ≤ 0.05 were written in bold font. The degree of freedom was seven.

Supplementary Table 6. Correlation tests between abundances of QS/QQ genes with environmental variables and predominant microbial orders [a]

Variable	LuxI		LuxR		AHL lactonase		AHL acylase	
	p-value	ρ	*p*-value	ρ	*p*-value	ρ	*p*-value	ρ
Depth	0.064801	-0.678	0.044786	-0.718	0.805679	0.104	**0.006535**	**0.857**
Temperature	**0.034542**	**0.743**	**0.017955**	**0.797**	0.820594	0.096	**0.018929**	**-0.793**
Salinity	0.313983	-0.409	0.279215	-0.437	0.135888	0.575	0.425638	0.329
DO (mg/L)	0.594343	-0.224	0.515668	-0.271	0.116769	-0.599	0.577428	-0.234
pH	0.051933	0.703	**0.032649**	**0.748**	0.808441	-0.103	**0.015874**	**-0.805**
PO_4^{3-} (μmol/L)	0.2403	-0.47	0.222753	-0.485	0.357387	0.377	**0.01682**	**0.801**
NO_3^- (μmol/L)	0.328908	-0.398	0.316755	-0.407	0.251831	0.46	**0.036824**	**0.737**
NO_2^- (μmol/L）	0.178173	-0.528	0.127302	-0.585	0.116674	-0.599	0.537927	0.258
NH_4^+ (μmol/L)	0.304359	-0.417	0.244474	-0.466	0.060224	-0.686	0.710936	0.157
SiO_4^{2-} (μmol/L)	0.202645	-0.504	0.182863	-0.524	0.39709	0.349	**0.013876**	**0.814**
BD7-8 marine group	**0.028016**	**-0.762**	**0.013026**	**-0.818**	0.288381	-0.429	0.13903	0.571
Candidatus Actinomarina spp	**0.042191**	**-0.724**	**0.017559**	**-0.798**	0.280293	-0.436	0.164226	0.543
Emiliania huxleyi	0.228895	0.48	**0.008869**	**0.841**	0.345534	-0.386	0.094171	-0.63
Phalacroma mitra	0.454963	0.31	**0.049169**	**0.709**	0.563133	-0.242	**0.017663**	**-0.798**

Pseudoalteromonas spp.	0.054604	0.697	**0.027742**	**0.763**	0.370347	-0.368	0.721375	-0.151
Alteromonas spp.	0.064186	0.679	**0.038391**	**0.733**	0.321275	-0.404	0.816839	-0.098
Thaumarchaeota spp.	0.10235	-0.618	0.092091	-0.633	0.708375	0.158	**0.018244**	**0.795**
Candidatus Nitrosopumilus spp.	0.344693	-0.386	0.277573	-0.438	0.248909	0.462	**0.043393**	**0.721**

[a] The *p*-values ≤ 0.05 were written in bold font. The degree of freedom was seven.

Supplementary Figures

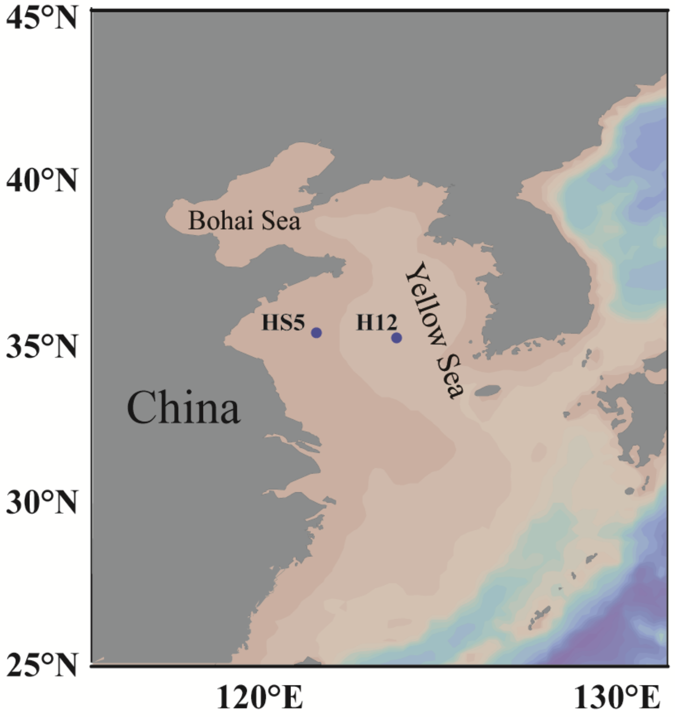

Supplementary Figure 1. Location of sampling sites in the Yellow Sea for POM collection.

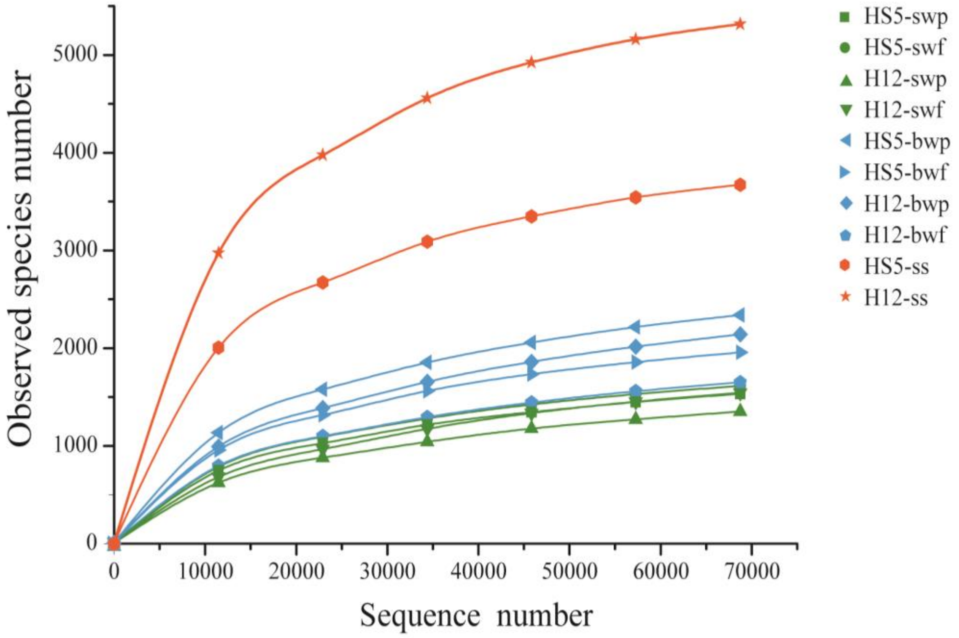

Supplementary Figure 2. Observed species number of each POM sample based on partial sequences of 16S rRNA gene.

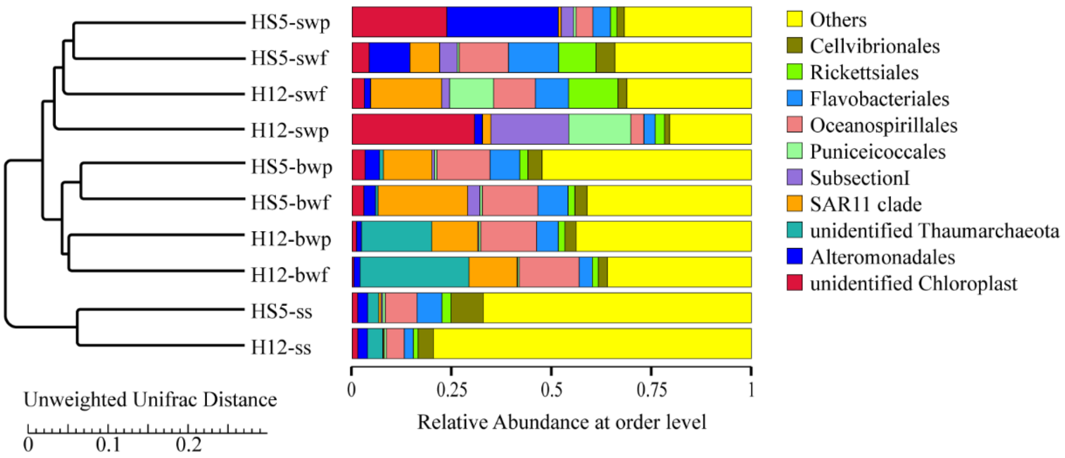

Supplementary Figure 3. UPGMA tree (left) and the relative abundance (right) of different microbial community structures in POM at order level.

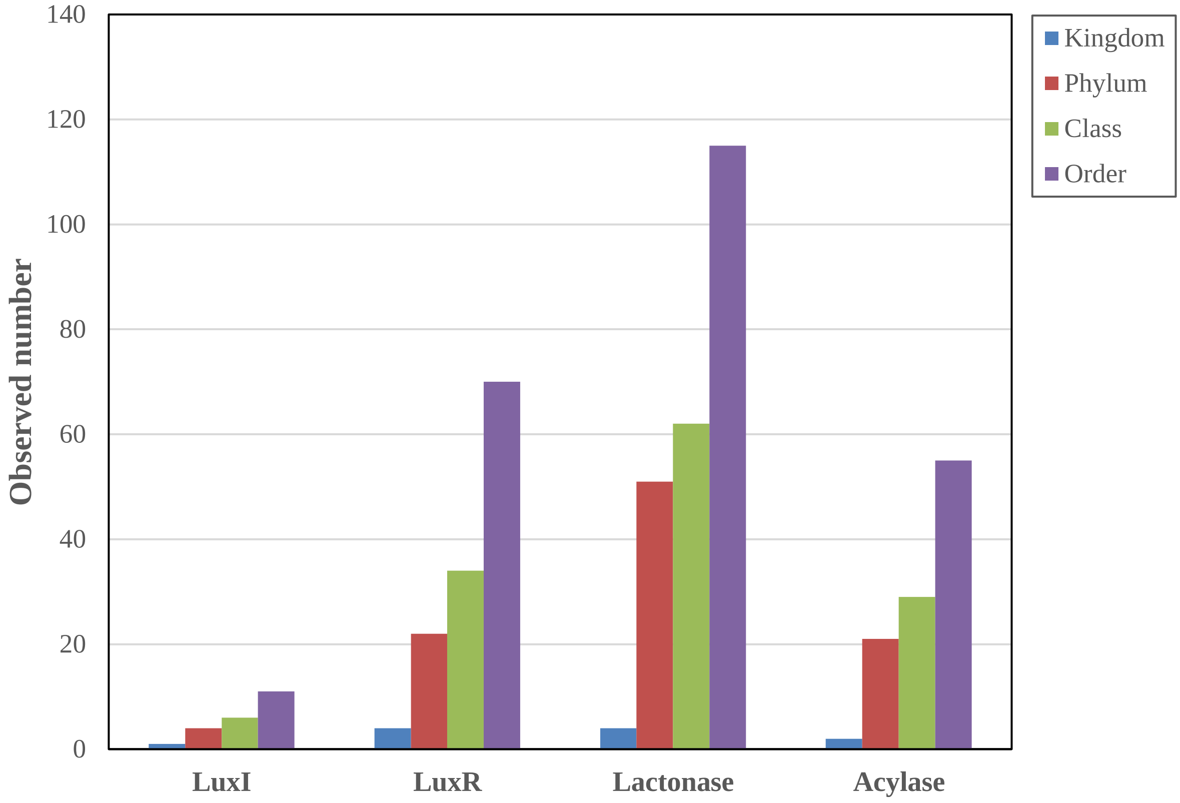

Supplementary Figure 4. Observed numbers of microbes possessing QS and/or QQ genes at kingdom, phylum, class, and order levels.

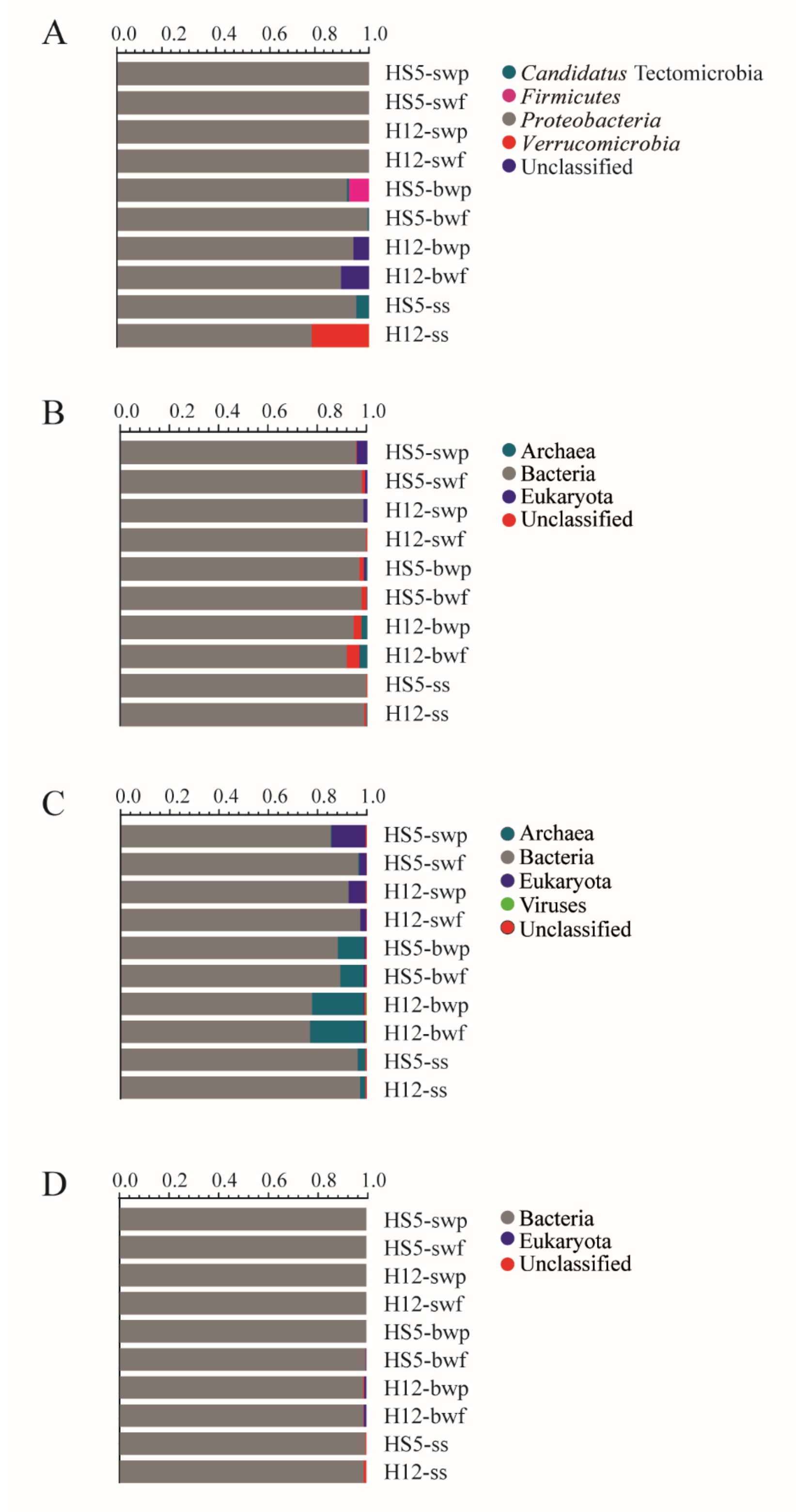

Supplementary Figure 5. Microbial assemblages possessing QS and QQ genes in POM. Panels A, B, C, and D represent genes encoding for AHL synthase, AHL receptor, AHL lactonase and AHL acylase, respectively.
